# Commentary: Reduced task-related quadriceps sEMG and limited functional transfer after 4-week low-load blood flow restriction training: a pilot randomized controlled study

**DOI:** 10.3389/fphys.2026.1842803

**Published:** 2026-07-08

**Authors:** Kevin Happ, Michael Behringer

**Affiliations:** Department of Sports Sciences, Goethe University Frankfurt, Frankfurt am Main, Germany

**Keywords:** arterial occlusion pressure (AOP), blood flow restriction (BFR), exercise induced muscle damage (EIMD), delayed-onset muscle soreness (DOMS), occlusion pressure prescriptions

We read with interest the recent pilot randomized controlled study by Xia et al. (2026), which examined task-related quadriceps surface electromyography (sEMG) and functional transfer after 4 weeks of low-load blood flow restriction (BFR) training. The authors provide valuable insights into training-induced changes in isokinetic performance and sEMG responses.

However, we would like to respectfully address a statement in the Discussion that does not accurately reflect the findings of our recent randomized controlled trial (Happ et al., 2025). In their Discussion (Section 4.5, p. 10), titled “BFR effectiveness and the role of individualized pressure”, Xia et al. write: “Recent evidence suggests that higher relative AOP can increase muscle damage and perceptual strain in some contexts (Happ et al., 2025)”. While we appreciate the authors’ intention to contextualize pressure-dependent responses, we respectfully wish to clarify that the direction of the effect reported in our study is opposite to that implied by this citation. Our single-blind randomized controlled trial (Happ et al., 2025) investigated the acute effects of four different AOP levels: no pressure (NP), low pressure (LP; 50% AOP), medium pressure (MP; 75% AOP), and high pressure (HP; 100% AOP) on exercise-induced muscle damage (EIMD) and acute physiological responses in 40 participants performing unilateral knee extensions at 30% of one-repetition maximum. Multiple outcomes were assessed at 1, 24, 48, and 72 h postexercise, with strength loss designated as the primary measure.

Across our four AOP conditions, the NP group (free-flow, no BFR) experienced the greatest strength loss at 24 h postexercise (mean [M]: − 8.1% ± 5.9%), while the LP group showed a comparable reduction. In contrast, the MP group (M = 1.9% ± 6.3%) and HP group (M = 2.4% ± 11.7%) demonstrated virtually no reduction in strength. Pain ratings followed the same pattern, with NP and LP reporting higher DOMS than MP and HP at 24 and 48 h postexercise. Applying the classification framework of Paulsen et al. (2012), the NP group exhibited mild-to-moderate EIMD, the LP group mild EIMD, and the MP and HP groups no EIMD. None of the secondary markers (creatine kinase, myoglobin, or muscle swelling) differed between groups over time. We therefore concluded that “BFR training, even at higher pressures, does not increase EIMD compared to free-flow exercise and that MP and HP may even attenuate strength loss and pain following exercise”. If anything, our data suggested a trend toward less muscle damage with increasing AOP, which we attributed to the protective effect of fatigue. By reducing susceptibility to contraction-induced dysfunction, fatigue may limit overstretching of sarcomeres and thereby prevent structural damage.

Accurate citation of safety-relevant findings is particularly important in the field of BFR training, where evidence on optimal pressure prescription is still emerging and practitioners depend on the literature to inform clinical and athletic decision-making. We are fully aware that Xia et al. addressed a different research question and did not assess muscle damage. Our aim is therefore not to contest their findings but to ensure that the AOP–damage relationship reported in our trial is accurately represented. We agree with the authors that further dose–response research on pressure and inflation strategies is warranted. Accordingly, accurate citation of the existing evidence is essential for guiding future research in this field.
